# A mixed methods analysis of clinics’ perspectives on community factors influencing access to medications for opioid use disorder

**DOI:** 10.1186/s13722-025-00643-1

**Published:** 2026-01-08

**Authors:** Samuel Jaros, Maryam Abdel Magid, Hannah Cheng, Michele Gassman, Hélène Chokron Garneau, James H. Ford II, Mark McGovern

**Affiliations:** 1 Department of Epidemiology and Population Health, School of Medicine, Stanford University, Alway Building, Mail code: 5405, 300 Pasteur Drive, Stanford, CA 94305 USA; 2 Stanford Center for Dissemination and Implementation, School of Medicine, Stanford University, Stanford, CA USA; 3 Department of Biomedical Informatics and Medical Education, UW Medicine, University of Washington, Seattle, WA USA; 4 Department of Medical Social Sciences, Feinberg School of Medicine, Northwestern University, Chicago, IL USA; 5 School of Pharmacy, University of Wisconsin - Madison, Madison, WI USA

**Keywords:** Mixed methods, Community attitudes, Clinic perspective, Opioid addiction treatment, Medications for opioid use disorder

## Abstract

**Background:**

How communities impact patients taking medication for opioid use disorder (MOUD) has not been well-studied. Understanding the experience of MOUD providers allows us to better understand and measure community attitudes toward MOUD and identify strategies to increase support.

**Methods:**

We deployed an explanatory sequential mixed methods design to analyze baseline data from the SITT-MAT clinical trial. Our quantitative instrument was seven Likert-scale questions asking about community attitudes toward MOUD analyzed through means, standard deviations, and principal components. The qualitative data were semi-structured interviews coded inductively using a thematic analysis. The quantitative and qualitative results were integrated to produce the findings.

**Results:**

We surveyed staff from 20 specialty care addiction and primary care clinics in Washington state as part of a larger clinical trial. Eleven sites were also selected to complete an interview. Participating clinics were primarily specialty addiction treatment programs (*N* = 14, 70%), outpatient (*N* = 17, 85%), and/or located in urban areas (*N* = 12, 81%). In the survey, participants most agreed that relationships with other clinics help provide better care and least agreed that system-level policies mandate MOUD. In interviews, some staff described how reliable relationships with other clinics improved employee morale and patient care while others added that loose collaborations had fallen apart, leaving patients without the care they need. Interviewees described how insurer and government policies have made it difficult to expand their MOUD offerings. The interview data also indicated community attitudes on MOUD have improved over time with some clinics using direct outreach to garner support for MOUD.

**Conclusions:**

Our results suggest that building local peer networks of clinics can improve staff morale and patient care in areas where community support for MOUD is low. Though system-level barriers to MOUD have been reduced, there is still room for improvement in simplifying reimbursements and funding for clinics looking to improve care. Our findings encourage further measurement of community attitudes toward MOUD and development of implementation strategies to build networks that support patients and clinics alike.

**Trial registration:**

The data for this study is from the Stagewise Implementation-to-Target – Medications for Addiction Treatment clinical trial registered as NCT05343793 on April 25, 2022.

**Supplementary Information:**

The online version contains supplementary material available at 10.1186/s13722-025-00643-1.

## Background

### Stigma in medication for opioid use disorder

High-quality, integrated, and accessible care for opioid use disorder (OUD) is essential for curbing the effects of the opioid overdose epidemic in the United States [1]. This care often takes place in specialty addiction and primary care clinics where patients build a relationship and rapport with their providers. Previous research has aimed to increase the quality, integration, and accessibility of OUD care by understanding how provider-patient stigma may play a part. One study educated providers in the best practices of helping those who may have OUD and found providers were significantly more likely to screen, diagnose, and refer patients with substance use disorder and implement harm reduction techniques post-initiative [2]. These findings were bolstered by systematic reviews examining the impact of provider stigma on patients. They catalogued problems in providers like perceiving medication for opioid use disorder (MOUD) as just another illicit substance, preference for abstinence treatment, and not knowing the benefits and risks of different MOUDs as consistent barriers [3–5]. Another intervention-focused review demonstrated effective techniques for reducing provider stigma towards patients [6]. While these studies implemented changes within clinics to improve care, truly effective implementation work must look outside the clinic to capture barriers to care that a patient might experience [7].

The methods to measure and change attitudes held by the wider community are less developed, even though the community is an important dimension in a multi-level conceptualization of addiction and addiction care [8]. Researchers have recognized the need to study the impact of decisions made outside the clinic by governments, businesses, and community members. One 2020 editorial outlined the theoretical links between how people talk about addictions and patients’ ability to start and continue treatment [9]. Another commentary emphasized that stigma is not “bound by the walls of the clinic” and requires holistic public health responses involving community members to support recovery [10]. This commentary contrasted with much of the literature on OUD stigma which focuses on the provider-patient relationship. In addition to seeing providers as potential sources for stigma, we can embrace the multi-level theory of addiction care and see them as allies in OUD treatment and examine how they and their patients are influenced by their surrounding communities [8].

### Clinics and communities

Some studies have begun to examine community stigma from the patient and community perspectives by identifying local factors influencing OUD care. Several studies within specific groups like rural Black patients, patients with HIV, and pregnant women have identified poor transportation and a fear of being judged by community members as detrimental to starting OUD treatment [11–13]. Other studies examining stigma from the community’s perspective noted splits in public opinion around the effectiveness of MOUD, whether people with OUD are dangerous, willingness to live in the same place as people with OUD, and support for harm reduction strategies like naloxone and syringe exchanges [14, 15]. However, there does seem to be a missing middle – the clinic perspective on stigma – which may shine some light on the groups that encourage or discourage OUD treatment. The staff in these clinics may be uniquely situated to comment on the long-term variability of community attitudes as they have often been working in the same location for years or decades. One study demonstrated the importance of the provider perspective on community, finding doctors were significantly less likely to screen for high alcohol consumption when community stigma was high [16]. Therefore, we believe that asking providers about community attitudes toward OUD care will allow us to identify groups of influence and describe how those groups affect treatment.

Simultaneously, there has been an increasing body of evidence demonstrating the effectiveness of community attitude interventions. Education interventions which focus on dispelling misinformation and spreading facts about effective interventions have been effective in reducing stigma against MOUD in providers and increasing knowledge about naloxone in college students [17, 18]. Capacity-building interventions which empower community members to be a part of the solution to the problems they see have been well-described and shown to be effective in stigma-heavy diseases like HIV [10, 19]. However, these studies primarily looked at stigma in general without examining what groups or strategies may be fruitful in reducing stigma.

### Objective and significance

We hypothesize that examining community attitudes from the OUD treatment clinic staff perspective will allow us to identify which community groups influence OUD care, differentiate how groups influence care, and propose strategies for improving MOUD availability in the future. The exploratory sequential study design of this study combines the statistical power of surveys with the depth of semi-structured interviews. This study also characterizes resistance and support as perceived by professionals working in OUD clinics, offering a unique perspective distinct from previous studies’ focus on provider-driven stigma. This approach can help determine which groups in the community help or hinder the clinic staff in providing MOUD services. For the purposes of this analysis, the “community” is anyone with an opinion on OUD care who can influence the participant clinics, providers, or patients politically or socially, usually within the clinic’s county. This definition allows us to focus on people who have a direct impact on the patients and providers in our study. Influences beyond this community we call “system-level factors”.

## Methods

### Data source

The data for this study was baseline data from the adaptive implementation trial Stagewise Implementation-To-Target – Medications for Addiction Treatment (SITT-MAT; NCT05343793; NIDA R01DA052975). Participants were clinics seeking to install, expand, and/or improve their MOUD services. The clinics included both specialty addiction and primary care clinics who see patients with opioid use disorder in Washington state. The specialty clinics could be residential or outpatient but could not be prescribing Methadone. As the trial progresses, clinics will receive progressively more intensive implementation strategies until they reach their goals [20]. The 28 SITT-MAT participating clinics who had enrolled in SITT-MAT before interview selection in April 2023 were eligible to be included in this analysis.

### Mixed methods design

This study employed an explanatory sequential mixed methods design where a quantitative survey was followed by qualitative interviews which explored deeper into topics covered by the survey. This design was intended to simultaneously gain a wider understanding of external factors influencing clinics overall and specific details on the experience of each clinic. The strengths of this schema granted the potential to make generalized claims about the experience of the participants, provide specific positive and negative examples of experiences, and suggest interventions for future studies based on what has worked for the participants in our study [21]. 

### Quantitative data

At enrollment, all participant sites were asked to complete the Integrating Medications for Addiction Treatment Index (IMAT) and the Inventory of Factors Affecting Successful Implementation and Sustainment (IFASIS) in addition to providing clinic characteristics and other study measures related to the care improvement aims of the trial. The IMAT and IFASIS are two widely-used measures with acceptable psychometric properties [22–24]. The IMAT can be found in Additional File 1, and the IFASIS can be found in Additional File 2. All seven questions mentioning “community”, “system level”, or “organizations in the geographic region” were included in the quantitative measures. The remainder of the questions from the surveys cover topics about clinical efficiency and perceptions about patients which were not covered by this study. Multiple employees including prescribers, nurses, counselors, and administrators at each clinic completed these surveys working as a group.

The primary analyses of the surveys consisted of descriptive statistics and principal component analyses. These methods allowed for easy comparison with the qualitative interviews. The IMAT and IFASIS survey question scales were standardized prior to the analyses [25]. The descriptive statistics consisted of a mean and standard deviation for each question and Cronbach’s alpha across all questions to examine agreement. The principal components analysis summarized the differences in survey responses into components that we interpreted as the primary axes of community support [26]. Principal components with Eigenvalues greater than 1 were retained. We also used Cronbach’s alpha to check question item agreement. All quantitative data were analyzed in R [27].

### Qualitative data

The completed survey results were used to select nine clinics for semi-structured interviews using a stratified sample across clinic variables like inpatient/outpatient, rural/urban, and high/low self-rated community support so that the extremes of each of these dimensions were represented in the interviews [28]. We also included two clinics who did not respond to the surveys to cover the widest range of experiences. The interview guide started with questions that ask for overall barriers and facilitators to providing addiction treatment and was revised for this study based on the quantitative results. As the most agreed with statement was about strong relationships with community organizations, a question about implementing outreach was added. A question asking about barriers to MOUD was expanded to include specific probes about system-level or policy barriers they have experienced based on the least agreed with statement. With the second principal component highlighting an important distinction between local and distant groups, questions were revised to specifically ask about what groups were supportive or detrimental to their ability to provide MOUD care. The full interview guide can be found in Additional File 3. The semi-structured interviews were conducted by MM and JF via Zoom over 30–60 min with one to five individuals for each clinic in April and May 2023. The interviewers were both male professors with PhD’s in clinical psychology and systems engineering, respectively. The interviews were recorded for audio and video, and participants were given a $25 gift card for their time.

SJ, MAM, HC, and MG transcribed and coded the interviews using a thematic analysis [29]. The codebook was developed inductively based on the interview guide and the interview transcripts, defining common codes across interviews [30]. We used two-part codes to describe the experiences in the transcripts. The first part encoded the valence of that experience: positive, negative, or mixed. The second part covered the different community groups identified in each experience. This section included codes for groups like businesses, law enforcement, other clinics, government, nonprofits, and schools. In this way, most excerpts where clinics spoke about a specific group received two codes: a valence and a group. The other codes included a section to cover non-specific experiences like system-level factors where the clinics talked about large-scale structural barriers and facilitators rather than specific people. These factors were coded separately because they extended beyond our local-focused definition of community. The full codebook with definitions is detailed in Additional File 4. To retain internal validity, two researchers coded the interviews independently and then met to resolve conflicts into consensus-coded interviews that were used in the analysis [31]. The coded excerpts were then grouped and categorized in relation to the quantitative results. These quotes were labeled as concordant, discordant, expansionary, or divergent compared to the survey results [32]. All qualitative data were analyzed in Dedoose [33]. In interview quotes displayed below, brackets indicate the excerpt was shortened, clarified, or censored for privacy.

## Results

### Study sample

The study sample consisted of 20 clinics of the 28 who were enrolled in SITT-MAT before we began selection for the interviews in April 2023. 18 clinics were included because they had completed the baseline surveys, and two additional clinics were included as interview-only participants to reduce internal bias. The remaining 8 clinics were excluded because they had not completed the baseline surveys and were not selected for an interview. The clinics varied in size from small clinics with only a few staff to larger clinic groups with multiple sites. Clinics in the sample were primarily substance use disorder clinics (*N* = 14, 70%) as opposed to primary care. Three (15%) of the clinics offered some kind of inpatient care. Most of the clinics were from urban areas (*N* = 12, 60%). After purposive sampling to achieve a diverse sample for the qualitative interviews, 11 (55%) clinics completed interviews. Clinic characteristics are included in Table 1.

**Table 1:** Clinic characteristics by mixed methods inclusion

Characteristic	All (*N* = 28)	Survey (*N* = 20)	Interview (*N* = 11)
Specialty N (%)			
Substance Use Disorder	19 (68%)	14 (70%)	8 (73%)
Primary Care	9 (32%)	6 (30%)	3 (27%)
Type N, %			
Inpatient	3 (11%)	1 (5%)	1 (9%)
Outpatient	23 (82%)	17 (85%)	8 (73%)
Both	2 (7%)	2 (10%)	2 (18%)
Urbanity N, %			
Rural (RUCC > 4)	5 (18%)	4 (20%)	4 (36%)
Suburban (RUCC 3–4)	4 (14%)	4 (20%)	2 (18%)
Urban (RUCC 1–2)	19 (68%)	12 (60%)	5 (46%)

RUCC – Rural-Urban Continuum Code

### Quantitative results

In the Cronbach’s alpha calculation for the quantitative survey, the questions achieved a 0.77 which suggested good agreement and the items all measured the same concept. Of the questions from the baseline surveys, the clinics most agreed with the statement “Because of our strong network relationship with other health and social service organizations in our community, we are better able to deliver MOUD” (Mean ± SD, 5.11 ± 1.08). Not only did this question have the highest agreement, but it also had the smallest standard deviation. The least agreed with statement with the lowest score was “System level policies mandate MOUD” (3.89 ± 1.84). This statement also had the largest standard deviation and was the only item where the mean score fell below 4, the neutral point on the 7-point Likert disagree to agree scale. Each statement, its mean, and standard deviation can be found in Table 2.

**Table 2:** Quantitative survey items and results

Source	Item Text	Scaled Mean (out of 7)	Scaled SD
IFASIS #3	A wide range of local community organizations (e.g., healthcare, social service, faith-based, government) are invested in and support the use of MOUD.	5.00	1.46
IFASIS #4	There is strong local, community advocacy for MOUD.	4.83	1.70
IFASIS #5	Community members or advisors have been and are regularly consulted on the overall fit and acceptability of MOUD from a diversity and inclusivity perspective.	4.17	1.47
IFASIS #6	System level policies mandate MOUD.	3.89	1.84
IFASIS #7	System level regulations ensure MOUD is available within organizations.	4.17	1.62
IFASIS #8	Because of our strong network relationships with other health and social service organizations in our community, we are better able to deliver MOUD.	5.11	1.08
IMAT #D5-6	Program leadership engages in regular meetings with other organizations in the geographic region to troubleshoot, improve communication and strengthen the network of care for patients on MOUD.	4.08	1.66

The principal components analysis gave further insight into the quantitative answers, revealing patterns in community attitudes towards MOUD. This analysis found the data grouped into two principal components. The first principal component loaded evenly and positively across all items, showing a plurality of the variance was captured by a single component representing positive or negative overall sentiment toward MOUD. The second principal component loaded positively onto IFASIS #3 and IFASIS #4 and negatively onto IFASIS #6 and IFASIS #7. IFASIS #3 and #4 asked about the “local community” while IFASIS #6 and #7 asked about the “system-level”. This second principal component seemed to suggest clinic perception differs based on proximity between the clinic and the community group in question.

### Qualitative results

The clinics described a variety of experiences across different community groups in the interviews. The only community group exclusively described positively was schools, where clinics mentioned collaborations in awareness and naloxone distribution. Clinics described businesses as helping carry naloxone, but also being the source of some negative stereotyping. Family and friends could be open-minded to MOUD or see it as replacing one drug for another. Some government bodies brought MOUD providers together and advocated for better treatment while others were simply a source of paperwork. Law enforcement and non-profits were assets to expanding MOUD availability with many clinics having tight collaborations, but a few clinics also described issues. The police were a particularly good ally in several communities where patients started treatment in jail and then continued with the same provider when released. Most clinics viewed peer clinics as strong collaborators for sharing methods and for getting patients access to additional resources except when poor communication led to patients being left in the dark. Other groups like political organizations and Facebook groups were mixed with some groups fighting for expanded resources and others attempting to make MOUD invisible. Exemplary positive and negative excerpts for each identified community group can be found in Table 3.

**Table 3:** Exemplary interview excerpt by community group and Valence

Group	Positive Clinic Experience	Negative Clinic Experience
Businesses	“We’re creating a project right now to provide outreach and education to area businesses on the use of naloxone, and when, to use it, what to do afterwards. Like especially libraries, convenience stores, McDonald’s.” – Administrator, Clinic A, SUD	[Who is posting negative comments about MOUD patients on social media? ] “I don’t know, half citizens and half downtown business owners?” – Administrator, Clinic B, SUD
Family and friends	“Nobody’s looking bad at [MOUD].You know, to be honest, I probably get more questions from the parents – ‘Why not?’” – Provider, Clinic C, SUD	“[Patients] come in, and it’s families don’t want them on it. So I think there needs to be a little bit more education to the family members that it’s okay for them to be on it.” – Provider, Clinic D, SUD
Schools	“We got a grant that was specifically focused for getting Narcan into schools, and so I do a lot of training, so we did 13 different schools in person and Zoom training for Narcan. We got over 300 kits of Narcan out to the community to the staff.” – Provider, Clinic B, SUD	None mentioned
Government	“We have [county] Behavioral Health and Recovery Division, and they form an umbrella over behavioral health organizations and agencies in the county, and they act as advocates to legislator. […] So [county], we have quite a few advocates that really will speak either on our behalf or help elevate our voices, which is really nice.” – Administrator, Clinic A, SUD	“About primarily 90% of our clients are Medicaid-funded. […] And so everything goes through [county]. They have very strict guidelines about not double dipping. […] We have to ask for a waiver, an exception, so that they can receive services from two different agencies. And it does…it…creates quite a barrier.” – Administrator, Clinic A, SUD
Justice and police	“Yeah, the [correctional] officers, probation officers make sure that they have the prescription, and I haven’t heard any probation officers say you can’t use your prescription.” – Provider, Clinic E, SUD	“[Sheriff] put signs up around the county, saying, ‘If you do drugs, we’re going to run you out of town.’ […] We’ve had a very difficult time getting MAT into the jail because they have a medical person [with a] ‘pull yourself up by the bootstraps’, and ‘you’ve made your bed now lie in it’ kind of mantra.” – Provider, Clinic F, PC
Nonprofits	“We have some local health advisory boards. I sit on one of the advisory boards myself, so I’ve kind of used that as a platform to address, like, you know, especially like housing and homelessness has been a big issue in our community. And then all that kind of goes hand in hand with, you know, with substance use and mental health.” – Provider, Clinic G, SUD	“We lose people sometimes like if they’re scheduled to come over here, and they’re a part of [nonprofit], and you know that other agency is supposed to be the one transporting them and bringing them to us, and then they drop the ball and can’t find them or forget to bring them then sometimes we may never hear from them again.” – Administrator, Clinic G, SUD
Other clinics	“We have a mental health facility right next door, which really helps with the warm hand offs back and forth. […] And sometimes they won’t even talk to a patient. They’ll just be like ‘Okay. You need to go here first and then come back to me when you’re not in complete withdrawal, and we can actually talk about your evaluation.’” – Provider, Clinic B, SUD	“Sometimes the communication wasn’t that great with some of the outside providers that we had just because they were running their own thing, and it was really all they cared about, was whether or not the patient was in counseling. They didn’t really communicate if they should seem to be struggling in a more severe way, or things of that nature.” – Administrator, Clinic H, SUD
Unofficial groups	“I just had an invitation from a local political group […]. They are trying to be across the aisle non-political and apolitical action type thing. And they’ve just invited me to sit on a panel about the fentanyl crisis. So you know, really seeing a big underlying push to address this in the community from a progressive and not from a ‘Oh, these people!’” – Administrator, Clinic I, PC	“There’s been a big thing going on in our community about the homeless, and to this group of our community being homeless equates to having a substance use disorder and behavioral health disorder. They are all one in the same to this community of people, and there’s like Facebook groups, and they go on there and just say horrible things about this population.” – Administrator, Clinic B, SUD

### Mixed methods results

Following the explanatory sequential mixed methods design, the qualitative results were analyzed in context of the quantitative findings. The comparisons between the quantitative and qualitative findings including meta-inferences on how the two compare can be found in Table 4.

**Table 4:** Joint table of quantitative and qualitative findings

Overall Finding	Quantitative Finding	Qualitative Findings	Mixed Method Meta-inferences
*Finding 1* Factors outside the clinic including insurers, laws, and poor infrastructure hinder patients from starting and continuing quality opioid addiction care. However, there are promising collaborations with law enforcement to divert people from jail or start treatment in jail.	“System-level policies mandate MOUD” is the least agreed-with statement in the quantitative battery of questions.	“About 90% of our clients are Medicaid-funded. […] So if somebody is receiving medication through one agency and they come to us for treatment, there is paperwork we have to fill out. We have to ask for a waiver, an exception, so that they can receive services from 2 different agencies. And it does…it…creates quite a barrier.” – Administrator, Clinic A, SUD	***Confirmation*** Policies do not reflect real-world needs
		“When you know somebody wants to go to treatment at, you know, 3 o’clock in the morning after they’ve been sexually assaulted, or you know, after they you know, have been kicked out of their house you know that’s the time that we need to…we need to be wrapping our arms around those people, and not saying, ‘well, we’ll see you on Monday’, or ‘we’ll see you in the morning’, because then we might not. […] We don’t have a lot of public transportation running all hours of day and night, either.” – Provider, Clinic G, SUD	***Confirmation*** Strategies to retain patients are lacking
		“When I’m looking at all these patients, and looking in their charts, and seeing all these folks with mental illness, and OUD mostly fentanyl use along with all these other you know, hearing voices, or you know, whatever their diagnoses are. […] I thought, man, those would be all the patients. We need to reach out, but you know they are just frequent fliers to these EDs and these other locations because they’re just discharged to the street.” – Provider, Clinic J, SUD	
		“I think more jails are […] even starting them on [Suboxone]. So we don’t see that barrier that we did maybe two years ago. So I do see an improvement in that. I see more of an acceptance of it. […] They’ve been on Subutex in jail, and then we just kinda continue it.” – Provider, Clinic D, SUD	***Discordance*** Diversion and MOUD in jail help patients recover
*Finding 2* While current quantitative measures capture important trends including overall support and local vs. system support, there are important domains revealed by the qualitative data that are missed by the quantitative questions including clinic outreach and how attitudes are changing over time.	The principal components analysis primarily loaded onto two factors which explain 69.1% of the variance. The first component, overall support, loads evenly and positively onto all questions. The second component, local vs. system, loads positively onto IFASIS 3 and 4 and negatively onto IFASIS 6 and 7.	“Our county prosecutor is one of the ones who really sees the problem with drug and minor crimes that he’s like, ‘Why should I prosecute these people when I know in my heart that’s not what they need?’ and he’s communicated a lot with our sheriff’s department. […] So you know, really seeing a big underlying push to address this in the community from a progressive and not from a ‘oh, these people!’” – Administrator, Clinic I, PC	***Confirmation*** Clinics experiencing support or hinderance from the community
		“The [tribal coalition] essentially pay for the facility. They’re running the upkeep of the facility. So we have real good rapport with that. […] They’re also not giving me any kickback when we’ve talked.” – Provider, Clinic C, SUD	
		“I would say there is not really any neighborhood or community support. […] When it comes to just the public as a whole, there’s still the stigma with it, and everything that comes along with buprenorphine. There’s a lot of people in the public that think that if you’re on a medication like Suboxone, that doesn’t mean that you’re actually clean.” – Administrator, Clinic H, SUD	
		“[Clinic staff member] has done a very good job of working with law enforcement to try and bring them on board. And by being in the jails, that really helps because it shows that we’re trying to help. You know it does help at the jail, because then people hopefully reduce their recidivism when they go back out into the community. It also helps the general population…in general they’re a better population, I think, to work with and, you know, if they’re getting their needs met.” – Provider, Clinic F, PC	***Expansion*** Outreach can improve the acceptance of MOUD
		“[Interviewer: ] Are there pockets of resistance that you have encountered? [Participant: ] I think, probably in the beginning, 7 years ago, when we were starting to talk about medication for opioid use disorder. […] We have been able to mentor, talk, discuss about ways to educate community and have an open discussion about what the fear is. And so I feel like we’re a pretty lucky area.” – Administrator, Clinic K, SUD	
*Finding 3* Local collaboration networks, especially with mental health clinics, provide more holistic care for patients. Local networks of clinics also provide collaboration and support when support from other groups is low.	“Because of our strong network relationships with other health and social service organizations in our community, we are better able to deliver MOUD” was the most agreed with statement in the quantitative battery of questions.	“So when we were working with one of the juvenile detention facilities in the area. Um…they had said, ‘Well, we can set up the Sublocade injections with one of the clinics here in town and that we would…’ they, that provider would continue to write for it so I could get around my policy issue, and then we would just facilitate her going down to get the shots every month.” – Provider, Clinic C, SUD	***Concordance*** Tight partnerships improve patient care.
		“I would say there is not really any neighborhood or community support. I mean, there is support within, you know, speaking with other agencies and such. But we all have the same goals. So there’s that kind of community that is very supportive.” – Administrator, Clinic H, SUD	***Expansion*** Networks of clinics create a social support group for providers and patients when the larger community is not supportive
		“Um we have [county provider network] that also um…a lot of us will go to different entities and kind of speak on the behalf of, you know, opiate use…um…convenings and things like that where it’s, we’re trying to get the word out and reduce that stigma around, this is part of treatment, this is part of the overall program, this is not continuing the criminal behavior.” – Administrator, Clinic A, SUD	
		“What can we do so that we can provide the medications because we have a lot of different agencies within the area but coordinating with them has been very difficult…like coordinating…we can refer so that our clients can get started on the medications, but then unless they’re under a certain court program, we’re not getting, you know, compliance reports. We don’t know when their medications change…we don’t know if they are not used.” – Administrator, Clinic A, SUD	***Discordance*** Weak partnerships lead to poor communication and patients not receiving the care they need.
		“So [site director] works, you know, pretty closely with other agencies in the area, and we have a couple who are…they drop the ball quite a bit. […] We kind of…we lose people sometimes like if they’re scheduled to come over here, and they’re a part of that program, and you know that other agency is supposed to be the one transporting them and bringing them to us, and then they drop the ball and can’t find them or forget to bring them then sometimes we may never hear from them again. We have no clue what happened to them. So that’s been a struggle. Very frustrating.” – Administrator, Clinic G, SUD	

The qualitative findings presented here are in addition to the qualitative findings presented in the text of the manuscript

MOUD – Medication for Opioid Use Disorder; OUD – Opioid Use Disorder; ED – Emergency Department

First, system-level factors outside the clinic and community including insurers, laws, and infrastructure remained barriers to patients starting and continuing MOUD. The clinics disagreed most with the statement “System-level policies mandate MOUD” in the quantitative survey. The interviews were largely concordant with this sentiment. One site struggled to get Medicare to cover the treatment their patients needed saying, *“Yeah*,* sure they can get medication*,* but if they need the counseling side of things*,* [Medicare’s] not going to cover it”* – Administrator, Clinic H, SUD. Another clinic experienced conflict with the local government, *“There was a clinic that opened up*,* specifically Methadone*,* and caused a big hu-ha. They pretty much put a moratorium on any further substance use treatment licenses in [city]*,* because um…it causes a really big issue with the community”* – Administrator, Clinic A, SUD. In discordant interview excerpts, some clinics mentioned system-level funding and policies supporting diversion to treatment and MOUD in jail. One clinic spoke about how state-level policies have opened new opportunities for improved treatment, *“So*,* our company got a grant specifically for the Blake Bill. […] And they can transport patients. They have a vehicle. They have a shower at their facility that people can use. They have a clothing closet. They have food vouchers. They have all kinds of stuff for the community to use”* – Provider, Clinic B, SUD. In this first mixed method finding, concordant quotes showed system-level issues still weigh on clinics’ ability to provide quality care, but there was some discordance when new funding opportunities enable clinics to expand and improve their care.

The second integrated finding produced four dimensions of community support. The principal components analysis found only two dimensions in the survey: one for overall support or lack thereof and a second for local community versus system-level actors. The qualitative data were concordant with these dimensions with excerpts describing positive and negative experiences at the local and system levels like, *“My multidisciplinary team really has a lot of participants from public health*,* from [company] counseling*,* from the Sheriff’s Department*,* from adult protective services”* – Administrator, Clinic I, PC and *“[A patient] was on Methadone a long time*,* and so he knows what resources are down in that area*,* and he said*,* there’s none. So I do know that lack of support is there”* – Provider, Clinic D, SUD. The qualitative data were also expansionary giving two additional dimensions. One clinic recognized their power to change community attitudes, *“But we eventually got in [the jail]*,* and we saw that one of our presentations*,* too*,* they gave us a hug. And I just said*,* I appreciate what you’re doing*,* you know*,* I know we’re on the same side”* – Administrator, Clinic F, PC. Several clinics also detailed community outreach projects that have decreased stigma for their patients. One clinic highlighted the importance of outreach in their work, *“So yeah*,* I do outreach to other organizations. Making sure that*,* uh*,* if the person is a father… I let the other social service agencies know that we’re here”* – Provider, Clinic E, SUD. The qualitative results were concordant with the quantitative, finding the same positive versus negative attitude and local versus system level dimensions. They were also expansionary, finding two additional dimensions: changes over time and clinic outreach.

The final integrated finding presented how collaboration with other clinics has been helpful, especially when community support was otherwise poor. The quantitative results found that the clinic’s network of community health and social service organizations supported their ability to provide MOUD. This sentiment echoed across the interviews. One clinic noted their collaborations supporting continuous care, *“We have one group […] They see them in detox and do the Zoom to kind of get on board with them. We then will refer them back to [collaborators] when they leave. […] They’re very good at getting them within three or four days of the call”* – Provider, Clinic D, SUD. Another clinic spoke about a consortium of clinics supporting each other *“But it was a very large program that was running for about five years*,* and up until COVID*,* has at least regular meetings*,* and they would do training. […] They’d have forums where we would talk openly about what is going on*,* or what we’re each noticing”* – Administrator, Clinic H, SUD. These supportive groups were especially important when the clinic did not otherwise have community buy-in, with another clinic saying *“I would say there is not really any neighborhood or community support. I mean*,* there is support within*,* you know*,* speaking with other agencies and such. But we all have the same goals. So there’s that kind of community that is very supportive”* – Administrator, Clinic H, SUD. However, some clinics spoke about loose collaborations when partners did not uphold their end of the deal which ended up hurting patients, *“Well*,* our director […] she works*,* you know*,* pretty closely with other agencies in the area*,* and we have a couple who are…they drop the ball quite a bit. […] the other agency is supposed to be the one transporting them and bringing them to us*,* and then they drop the ball and can’t find them or forget to bring them then sometimes we may never hear from them again”* – Administrator, Clinic G, SUD. These concordant results emphasized the importance of fostering strong, reliable, and trusting relationships between treatment providers and complementary organizations for the clinic’s, the staff’s, and the patients’ benefit.

## Discussion

### Research objectives and findings

This study explored the relationship between clinics that provide medication for opioid use disorder and their surrounding communities. We found the clinics experienced stigmatization and support from a variety of community groups. Overall, the clinics positively rated peer clinic support and rated system-level factors as less supportive in the quantitative surveys. The interviews added that these system-level factors hindering care tended to be laws and bureaucratic structures like reimbursements that make their work and their finances more difficult. Our analysis of the different dimensions of community support revealed changes over time and clinic outreach as important factors for future surveys to consider. The quantitative and qualitative data concurred with the importance of consistent, trusting collaborations across clinics in maintaining a continuum of care and treating the underlying causes of OUD.

The concordant integrated finding that system-level barriers still exist and weigh on clinics’ ability to deliver care is particularly actionable. Over the last two decades, we have taken strides toward ensuring access through laws mandating MOUD coverage like the Mental Health Parity and Addiction Equity Act in 2008 and the Affordable Care Act in 2010. The Consolidated Appropriations Act, 2023 further reduced barriers to MOUD access by removing the X-waiver requirement for MOUD prescribers. However, our data suggest that the bureaucratic and paperwork burdens from the system-level remain a particular pain point. Reducing red tape surrounding MOUD; incentivizing simplifications in prescribing and paying; and directly funding those working to improve care are important targets for future legislation [34–36]. Our one discordant finding where the interviews showed system-level factors helping clinics was a funding opportunity resulting from Washington SB 5536. This law primarily made possession of drugs illegal again after *State v. Blake*, but it also outlined a pathway to fund diversion programs so that individuals charged with simple possession could have their charges dropped by meaningfully engaging with a treatment program [37, 38]. Expanding this incentive elsewhere could improve induction and retention on MOUD. Further examination of laws like these could also reveal meaningful strategies where laws create pathways not paperwork, giving more patients an onramp into treatment.

Our second integrated finding identified several ways to improve how future research conceptualizes and measures MOUD stigmatization. We saw concordance across methods in the positive versus negative attitudes and local versus system level groups dimensions. These results encourage future studies to continue asking directly which community groups help and hinder care. This finding also suggests that a multi-level conceptualization of addiction best models the individual, community, and societal factors that lead to addiction and hinder recovery [8, 39]. We also found that the qualitative results expanded on the quantitative with the additional dimensions of changing attitudes over time and clinics performing outreach to reduce stigma. Understanding that attitudes have been changing over relatively short periods of time is important to account for in longitudinal trials. The outreach dimension can be implemented directly through capacity-building in law enforcement partnerships that emphasize common goals of reducing recidivism and improving health, which other studies have found fruitful [40–44]. Our findings outline the importance of examining and acting on the many dimensions of community stigma that patients and providers experience.

Our third finding indicated that the most fruitful relationships for staff and patients seemed to be local clinic networks that expanded access to mental health care and social services. This finding encourages investment in implementation strategies that create networks of clinics and nonprofits in the same physical area. These networks create social opportunities for providers, cross-refer patients for additional care, and promote collaborative networks rather than hierarchical structures where failures at the central node cascade out [45–47]. Our findings suggest that partnerships like the Massachusetts Collaborative Care Model where expertise is spread across sites reduce provider burden and may result in more referrals and longer retention [48]. In addition to providing better care, social networks of healthcare providers can help prevent physician burnout. Our findings contribute to a growing body of literature that recommends investment in mitigating moral injury and career dissatisfaction across healthcare providers [49]. We believe that shifting focus toward building mutually beneficial peer relationships between clinics will build a stronger health care system that better addresses patients’ needs.

Our findings provide a solid foundation for future work to reduce system-level barriers, improve the measurement of community attitudes, and develop local collaborations that improve MOUD access. Future studies should continue to explore how community attitudes affect MOUD prescribing. These studies should incorporate the dimensions of community support and work to build collaborations across clinics. Policies should continue to make MOUD prescribing easier by decreasing the burden placed on the clinics. Other opportunities that start people on MOUD, increase referrals to mental health and other care, and collaborate with law enforcement should be a high priority for those looking to curb the effects of opioid addiction in their communities.

### Study strengths and weaknesses

This study implemented methods to maximize strengths while minimizing weaknesses. It was situated within the long-term intensive clinical trial SITT-MAT which greatly increases the clinics’ investment in the study and trust of the clinic staff. The interviewees provided a wealth of detail to our measurements with their experience and expertise. Being part of a larger trial also allowed this study to achieve a relatively large sample size as clinics were incentivized to participate by the coaching they receive later in the trial. The mixed methods explanatory sequential design was also a primary strength. This design is highly robust, combining the generalizability of a survey instrument with the in-depth nature of the qualitative interviews. The purposive sample ensured the interviewed clinics represented diverse perspectives. However, this study sample resided entirely within one, relatively democratic state. Some of the community strategies suggested may not be as successful in a different political climate. To participate in SITT-MAT, the clinics also needed to have the spare time to dedicate to data collection and coaching. Therefore, it is likely the clinics in the study were more well-resourced and less over-burdened than clinics in general. The eight clinics excluded from our study due to incomplete surveys could also have had divergent community experiences which we were unable to measure. Our analysis also treated clinics equally regardless of their size or service area. We were unable to control the number of staff members at each clinic that participated in the survey or interview. These limitations should be considered before applying our results to a different population as clinics may not have the same bandwidth for education, outreach, and collaboration.

### Significance

This study sets the groundwork for future exploration into specific groups’ attitudes toward MOUD and future implementation trials building collaborations to improve MOUD care. We have shown that providers’ perceptions of community attitudes are quantifiable and worth measuring in future studies. Our results also identified clear and actionable targets for stigma reduction implementations like creating networks among law enforcement, public health officials, mental health clinics, nonprofits, and MOUD clinics. By acting upon these opportunities, policy work can build out an infrastructure designed to treat people with opioid use disorder holistically. Accurately measuring and addressing community stigma will expand the availability of MOUD and empower community members to be a part of the solution to opioid addiction.

## Conclusion

We set out to measure community attitudes toward MOUD from the clinic perspective using the strengths of an explanatory sequential mixed methods design. We found that, overall, staff had both positive and negative experiences with most community groups. Interactions with insurers and funding organizations were primarily negative with bureaucratic systems preventing them from providing needed care. Reconsidering the required approvals and paperwork for funding and reimbursements can help providers dedicate more of their time to patients. After integrating our quantitative and qualitative results, we found at least four dimensions to community attitudes: positive versus negative; local versus system-level; change over time; and clinic outreach. Future studies and interventions on community attitudes should measure at least these dimensions. The clinic outreach dimension should be of specific interest where outreach to law enforcement leads to MOUD availability in jails. We also consistently found that peer-based collaborations with nearby clinics improved the patient and provider experience. Future work aiming to build capacity in addiction treatment should take these naturally built collaborations into account in their design. Overall, our study demonstrates the importance of examining community attitudes. Our data also show that the clinic staff have an important perspective beyond the patient experience which is worthy of study. With interventions building collaborations across clinics, nonprofits, and law enforcement, we can begin to break down MOUD stigma, have patients start and stay on treatment, and ensure MOUD patients are receiving all the care they need.

## Supplementary Information

Below is the link to the electronic supplementary material.

Supplementary Material 1

Supplementary Material 2

Supplementary Material 3

Supplementary Material 4

## Data Availability

The data analyzed in this study are not publicly available due to the ongoing nature of the larger clinical trial and due to identifiable details contained in the full data sets. However, these data can be made available on reasonable request to the corresponding author.

## Abbreviations

HIV: Human Immunodeficiency Virus
IFASIS: Inventory of Factors Affecting Successful Implementation and Sustainment
IMAT: Integrating Medications for Addiction Treatment
IRB: Institutional Review Board
MOUD: Medication for Opioid Use Disorder
NIDA: National Institute on Drug Abuse
OUD: Opioid Use Disorder
RUCC: Rural-Urban Continuum Code
SB: State Bill
SITT-MAT: Stagewise-Implementation-To-Treatment – Medication for Addiction Treatment
