# Supplementary material for: A mixed methods analysis of clinics’ perspectives on community factors influencing access to medications for opioid use disorder
Source: Addict Sci Clin Pract. 2026 Jan 8;21:10. doi: 10.1186/s13722-025-00643-1 (PMC12825234; doi:10.1186/s13722-025-00643-1)
Supplement: Supplementary file 1 — Supplementary Material 1 [file 13722_2025_643_MOESM1_ESM.docx]

**Additional File 1:** Integrating Medications for Addiction Treatment (IMAT) Index Survey Instrument

Respondents select one of the following answers for each item:

1. Not Integrated
2. Between 1 and 3
3. Partially Integrated
4. Between 3 and 5
5. Fully Integrated

Infrastructure

D1-1. Senior agency and program leadership, including CEO, CMO, board and clinical directors, strongly support providers prescribing MOUD in the program

D1-2. Medical records and releases of information are privacy compliant with 42CFR and HIPAA regulations

D1-3. Insurers cover medical consultations and visits for management of MOUD or medical services are covered by bundled contractual rates

D1-4. Insurers cover MOUD (buprenorphine and naltrexone IM) or MOUD are covered by bundled contractual rates

D1-5. Insurers cover general medical services

Clinic Culture and Environment

D2-1. All program staff accept and welcome equally persons with OUD—no evidence for stigma or discrimination

D2-2. Open display and distribution of patient informational materials about OUD and MOUD in common areas therapy rooms and offices

D2-3. Patients and services are visibly integrated in general clinic spaces and in routine operations

D2-4. All program staff believe offering MOUD to patients in this program is appropriate

Patient Identification and Initiating Care

D3-1. All new and existing patients are screened using a standardized universal measure for opioid use risk

D3-2. All patients who screen positive receive a standardized indicated assessment and, if positive, an OUD diagnosis is made and documented

D3-3. All patients seen in this program on dosages of >90 mg of morphine equivalents (MMEs) for >3 months to manage chronic non-cancer pain are reviewed and evaluated for potential OUD diagnosis and appropriateness for buprenorphine

D3-4. A protocol for identification, diagnosis and treatment initiation exists for conditions commonly comorbid with OUD including other substance use disorders

D3-5. A protocol for identification, diagnosis and treatment initiation exists for conditions commonly comorbid with OUD including other psychiatric disorders such as depression, anxiety, PTSD or other mental health problems

D3-6. A protocol for identification, diagnosis and treatment initiation exists for infectious disease commonly comorbid with OUD, including HIV and HCV

D3-7. For patients diagnosed with OUD, the prescription drug monitoring clinic (PDMP) is queried

D3-8. For patients diagnosed with OUD, a point-of-care toxicology test is performed, i.e. urine drug screen, with built-in and/or rapid on-clinic immunoassay testing

D3-9. Patients with OUD are presented with clear treatment options, patient preferences are discussed, and a shared decision-making approach used

D3-10. Criteria for offering MOUD in the clinic are clear, they are documented in policy, patient information sheets/brochures and consent forms, and they are highly inclusive

D3-11. Three care components are performed for all patients using MOUD: Withdrawal symptoms are evaluated, side effects are discussed, and comfort medications to treat opioid withdrawal are made available

D3-12. Patients choosing MOUD, either buprenorphine or naltrexone long-acting injection, can be started on medication within 72 hours

D3-13. The program has a patient treatment agreement document that describes expectations of the program and of the patient on MOUD

D3-14. Using a protocol clear to both staff and patients, eligible patients can start the MOUD either at home or onsite at the program

Care Delivery and Treatment Response Monitoring

D4-1. Patients started on MOUD have at least 1 follow-up visit within 14 days (2 weeks)

D4-2. Patients started on MOUD have at least 2 follow-up visits within 30 days (1 month)

D4-3. Ongoing toxicology testing, i.e. urine drug screen, is performed at least monthly, at random, and observed

D4-4. The prescription drug monitoring clinic (PDMP) is queried at least bi-monthly

D4-5. A protocol exists for random pill or film counts for patients prescribed buprenorphine

D4-6. A protocol exists, based on treatment response—including toxicology results and patient report of functioning—to adjust dose, frequency of visits and toxicological monitoring

D4-7. A systematic approach (e.g., ASAM criteria) is used to assess patient functioning and social determinants; This approach supports treatment planning which may include additional physical or behavioral health services either within this clinic or offered in another setting

D4-8. A systematic approach, such as the ASAM criteria or Treatment Needs Questionnaire, is used to determine need for a more intensive level of care (residential, hospital) or setting (methadone clinic)

D4-9. Patients are neither encouraged nor required to taper or discontinue MOUD after a certain period of time or once stabilized or with improved functioning

D4-10. Six-month retention rates of patient panel on MOUD are tracked to examine this clinic’s processes

Care Coordination

D5-1. The program uses a team based care approach to manage patients treated with MOUD; team members may include physicians, nurse practitioners, physician assistants, nurses, behavioral health clinicians or counselors, peer specialists, or pharmacists; and with clearly defined, written roles and responsibilities for each member of the team

D5-2. A registry of patients on MOUD is used to track patient attendance, visit planning and treatment response

D5-3. With the most common health care and social service partners, the program has memoranda of understanding, agreements or clear understanding of methods to coordinate care, accept referrals, refer or link patients with primary care and/or specialists (e.g. addiction, psychiatry, OB/GYN) or services (e.g. DCFS, probation and parole)

D5-4. The program has a 42CFR and HIPAA compliant set of forms to exchange or release clinical information with patient consent

D5-5. An assertive community outreach procedure exists for patients who have not made appointments or about whom there is clinical concern (phone or home visit)

D5-6. Program leadership engages in regular meetings with other organizations in the geographic region to troubleshoot, improve communication and strengthen the network of care for patients on MOUD

Workforce

D6-1. X-waivered prescriber(s) onsite to prescribe MOUD

D6-2. Nursing or pharmacist personnel are onsite to manage MOUD and nursing-related needs of patients; a nurse or pharmacist care manager model is used to perform activities during patient visits either in individual or group formats; there is coordination of care with other health care providers; patient and family education is provided

D6-3. Licensed behavioral health clinician(s) with credentials in both mental health AND addiction assessment and treatment are onsite; have expertise to conduct evaluations, individual, group and family/couples therapies; there is expertise in integrated behavioral health and in team-based primary care; either individual behavioral health clinicians have expertise in both mental health and addiction OR two or more clinicians have combined expertise

D6-4. Staff or volunteer affiliation with peer recovery support group network (e.g. NA, AA, MA, Al-Anon) to educate and connect patients on MOUD and their support persons to these resources

D6-5. Administrative support to manage registry, coordination of care, liaison with other agencies, and funders

Staff Training and Development

D7-1. X-waivered providers/prescribers and other clinicians are actively involved in CME or equivalent continuing education and other advanced learning opportunities focused on MOUD, addiction and integrated behavioral health care

D7-2. All non-clinical staff, including administrative and support personnel, have basic training in MOUD AND substance use disorders and treatment their treatment

D7-3. All staff (clinical and non-clinical) have completed training in empathy and stigma reduction for persons on MOUD AND substance use disorders and their treatment
