# Supplementary material for: A mixed methods analysis of clinics’ perspectives on community factors influencing access to medications for opioid use disorder
Source: Addict Sci Clin Pract. 2026 Jan 8;21:10. doi: 10.1186/s13722-025-00643-1 (PMC12825234; doi:10.1186/s13722-025-00643-1)
Supplement: Supplementary file 2 — Supplementary Material 2 [file 13722_2025_643_MOESM2_ESM.docx]

**Additional File 2:** Inventory of Factors Affecting Successful Implementation and Sustainment (IFASIS) Survey Instrument

Respondents select one of the following answers for each item:

1. Strongly Disagree
2. Disagree
3. Somewhat Disagree
4. Neutral
5. Somewhat Agree
6. Agree
7. Strongly Agree
8. Does Not Apply

System Level

1. MOUD has strong support from system-level leadership (public health: federal and state agencies; health system, chief officers).
2. MOUD is a service or a benefit financially covered by public and private reimbursement mechanisms.
3. A wide range of local community organizations (e.g., healthcare, social service, faith-based, government) are invested in and support the use of MOUD.
4. There is strong local, community advocacy for MOUD.
5. Community members or advisors have been and are regularly consulted on the overall fit and acceptability of MOUD from a diversity and inclusivity perspective.
6. System level policies mandate MOUD.
7. System level regulations ensure MOUD is available within organizations.
8. Because of our strong network relationships with other health and social service organizations in our community, we are better able to deliver MOUD.
9. There is widespread consensus among staff and leadership about the value and benefits of MOUD.

Program Level

1. Our leadership has removed obstacles to delivering MOUD.
2. Our leadership supports continuous learning.
3. Our leadership has a long-term strategy and commitment to explore, implement, and spread MOUD.
4. Our leadership consistently champions and engages staff and teams to improve care.
5. Our leadership carefully monitors and holds the providers accountable for delivering MOUD.
6. There are enough qualified staff, who with training, will be able to deliver MOUD.
7. MOUD will not require the hiring of a new or different type of staff member.
8. Patient care staff have input into whether MOUD is offered in the organization.
9. Our leadership and/or staff are from or reside in the communities that we serve.
10. Our organization has sufficient resources such as staff, time, funds, and materials to implement MOUD.
11. Internal polices exist that make MOUD easy to deliver.
12. MOUD will require a new set of policies.
13. MOUD can be readily incorporated into our workflow.
14. Our program collects and examines patient data by demographic or other social indicators to identify potential inequities in delivery of MOUD, and shares and troubleshoots results.
15. MOUD is expensive for our organization.

Staff Level

1. MOUD is worthwhile.
2. MOUD is easy to use.
3. Most staff will learn to use MOUD very quickly.
4. Implementing/Expanding MOUD seems doable for our team.
5. Our staff, with training and consultation, will be able to deliver MOUD easily.
6. As a group, our staff are flexible and adapt quickly to new opportunities.
7. Our staff are generally open to change and new ways of doing things.
8. Our staff are easily reachable and responsive.
9. Our staff take the time to answer questions about MOUD.
10. Our staff are mindful of patients’ values and beliefs.
11. Our staff try to accommodate patients’ values and beliefs.
12. Our staff are racially/ethnically diverse and includes people with live experience that reflect our patient population.

Patient Level

1. Patients can afford MOUD.
2. MOUD meets patients’ needs.
3. Patients are asking for MOUD.
4. Patients are reluctant to engage in MOUD.
5. MOUD is beneficial to all patients regardless of social determinant characteristics, such as race/ethnicity, gender, and income.
6. MOUD can be tailored and delivered in a way that fits patients’ cultural beliefs.
