# Supplementary material for: A mixed methods analysis of clinics’ perspectives on community factors influencing access to medications for opioid use disorder
Source: Addict Sci Clin Pract. 2026 Jan 8;21:10. doi: 10.1186/s13722-025-00643-1 (PMC12825234; doi:10.1186/s13722-025-00643-1)
Supplement: Supplementary file 3 — Supplementary Material 3 [file 13722_2025_643_MOESM3_ESM.docx]

**Additional File 3:** Qualitative Interviews Codebook

| **Code** | **Descriptor** |
| --- | --- |
| Community Support | This code group includes responses for the question regarding community groups that are supportive or not supportive of OUD care in their community. All excerpts in this section should receive at least two codes: 1 affect (positive/negative) and 1 community group |
| - Mixed Opinion | All community support excerpts that express mixed opinion |
| - Negative Opinion | All community support excerpts that express negative opinion |
| - Positive Opinion | All community support excerpts that express positive opinion |
| - Community Outreach | Specific actions taken by the clinic to engage with the community around the clinic. This code describes events, talks, and campaigns to spread awareness and understanding of medication for opioid use disorder. |
| - Stakeholders | These codes specify community groups that should be co-coded with an opinion (positive, negative, mixed). |
| - Businesses | This code is for any for-profit, named group. These must be incorporated entities that primarily engage for payment. This group includes chambers of commerce. |
| - Family/friends | This code is for first-degree relatives and friends of patients within the study sites. These people must interact directly with patients and have a relatively close relationship with them. |
| - Generalized feelings | This code is for when the interviewee expresses what they believe are the general feelings of the generalized community. This code only applies if they seem to be generalizing rather than talking about a specific group. A specific group that is unnamed would NOT apply, instead being coded as “group not specified”. |
| - Government | This code is for any group that has power in an official capacity. Some examples of these organizations include HOAs, city, county, state, or federal governmental groups. This code includes elected and appointed officials. This code does NOT include tribal governments. |
| - Group not specified | This code is only for when the speaker is talking about a specific group, but they do not elaborate on what the group is. If the speaker is generalizing the feelings of the community overall, it would NOT apply, instead being coded as “generalized feelings”. |
| - Law enforcement | This code includes all law enforcement groups including police, sheriff, parole officers, and jail/prison staff. This group also includes people proximate to these people such as spouses as long as they are operating as relatives of law enforcement officials. |
| - Nonprofits | This group includes all formal, named, non-profit groups who act for a specific goal. This group includes tribal groups, religious groups, and harm reduction suppliers. These groups cannot provide health care directly as they would be "other clinics". |
| - Other clinics | All other health care providers who are not part of the study. Examples of this group include hospitals and mental health centers. These other clinics may be collaborators, competitors, or both to the participant site. |
| - Schools | These are any formal education centers. This group includes parents when interacting directly with the school. This group also includes individuals within the school system like teachers, principals, school nurses, superintendents, and district officials. |
| - Unofficial groups | These are groups that exert informal power. They are loosely associated, like a Facebook group. This group serves as a catch-all for organized groups that do not apply to another code. These groups must be at least loosely organized or else they would fall into “generalized feelings”. |
| Site Barriers | These are negative aspects of the participant site, according to the interviewee. This code does NOT include positive or negative aspects of the SITT-MAT study. |
| - Systems level | This code includes all actors external to the participant site including financers, governments, and community groups. |
| Site Facilitators | These are positive aspects of the participant site, according to the interviewee. This code does NOT include positive or negative aspects of the SITT-MAT study. |
| - Systems level | This code includes all actors external to the participant site including financers, governments, and community groups. |
| Good Quotes | Any excerpts that are quote-worthy for future presentations and publications |
| Health Equity | This code applies to any mention of a different experience of addiction or addiction treatment among subgroups. This code can be applied alongside any other code or on its own. This code is NOT for merely improving care overall, but for specific mentions of different care, treatment, or experience among subgroups of patients that may be due to an injustice. |

All child codes inherit their parent codes as indicated by indenting and coloring.

OUD – Opioid Use Disorder

HOA – Homeowners Association

SITT-MAT – Stepwise Implementation To Treatment – Medication for Addiction Treatment
