# Supplementary material for: A mixed methods analysis of clinics’ perspectives on community factors influencing access to medications for opioid use disorder
Source: Addict Sci Clin Pract. 2026 Jan 8;21:10. doi: 10.1186/s13722-025-00643-1 (PMC12825234; doi:10.1186/s13722-025-00643-1)
Supplement: Supplementary file 4 — Supplementary Material 4 [file 13722_2025_643_MOESM4_ESM.docx]

**Additional File 3:** Semi-structured Qualitative Interview Guide

**SITT-MAT Key Informant Interview Guide**

**GROUP (STAFF) and INDIVIDUAL (LEADER) VERSIONS**

**Baseline/Year 1**

***[Group interview only]*** *Let’s get started by introducing ourselves one at a time. Name and role at (NAME OF PROGRAM/CLINIC).*

***[Individual interview only]*** *Tell me about your role in the organization and on this project specifically.*

- **Site Name and Characteristics:**
- **Role(s) of Interviewee(s):**

1. ***What are you hoping to get from participating in SITT-MAT?***
2. ***What kinds of challenges or barriers has (NAME OF PROGRAM/CLINIC) experienced while implementing, improving, or expanding your MOUD services?***
   1. [Probe] *Do you anticipate any additional challenges?*
   2. Probe for challenges across system-, clinic-, staff-, and patient-levels
3. ***What kinds of strategies or facilitators have helped (NAME OF PROGRAM/CLINIC) to implement, improve, or expand MOUD services within your program?***
   1. [Probe] *What do you anticipate as additional facilitators?*
   2. Probe for challenges across system-, clinic-, staff-, and patient-levels
4. ***Within your county, how would you describe your community’s overall support for (NAME OF PROGRAM/CLINIC)’s MOUD services?***
5. ***Who is particularly supportive or not supportive in your community?***
6. [Probe] *What makes you say this?*
7. Probe for actors and actions of community players. Community players can be at the county level or lower that are supportive or not supportive. Potential starting places are government bodies, neighborhood residents, and religious groups.
8. ***What actions have you taken to get community buy-in for the work (NAME OF PROGRAM/CLINIC) is doing with respect to treating people with OUD using MOUD?***
9. ***In terms of the SITT-MAT project, how would you describe your experience overall?***
10. [Probe] *Are there things that have been particularly helpful or not helpful?*
11. [Probe] *What would you want more or less of?*
12. ***As you know, we have organized this project so you only get the support that you need. At this juncture, the first line of support is data that we collect and then share with you by creating dashboards. How has this part been going for you?***
13. [Probe] *Has it been helpful or not helpful?*
14. [Probe] *Any suggestions for making it more useful/impactful?*
15. ***There are additional implementation supports on the horizon for the future. How do you feel about having to wait for these supports and not having them available now?***
16. ***We are always curious if there have been surprises in projects like these. Over the last 12 months…***
    1. ***What positive or negative internal factors have happened that you may not have expected?***
       1. [Probe] *What is their impact?*
    2. ***What positive or negative external factors have happened that you may not have expected?***
       1. [Probe] *What is their impact?*
17. ***As we wrap up, I want to be sure that we’ve covered the key items. But just to be sure, are they any thoughts you have or have been mulling over quietly that we did not get to that you would be open to sharing? Anything else?***

***We are really grateful to you for making the time, and for taking the time to talk with us. We know how busy you are and how you have many competing priorities.***
